# Attachment and Executive Functions in ADHD Symptomatology—Independent Inputs or an Interaction?

**DOI:** 10.3390/brainsci10110765

**Published:** 2020-10-22

**Authors:** Łukasz Konowałek, Tomasz Wolańczyk

**Affiliations:** Department of Child and Adolescent Psychiatry, Medical University of Warsaw, Żwirki i Wigury 61, 02-091 Warszawa, Poland; twolanczyk@wum.edu.pl

**Keywords:** ADHD, executive function, attachment

## Abstract

Despite a multitude of research on executive functions and attachment in Attention Deficit Hyperactivity Disorder (ADHD), a synthetic approach to the matter seems to have been lacking. In this review, we attempt to check the available data against two hypotheses: (1) executive functions and attachment influence ADHD symptoms independently; (2) executive functions and attachment interact to produce ADHD symptoms. We found no evidence falsifying the former hypothesis and some evidence to refute the latter. Limitations of current study approaches and suggestions for further research are discussed. Specifically, we propose an attachment representation, rather than attachment style, approach to measuring the mediation thereof between executive dysfunctions and ADHD.

## 1. Introduction

Attention Deficit Hyperactivity Disorder (ADHD) is a psychiatric condition that contains a cognitive deficit (attention disorder) in its name. It is therefore no wonder that it has been investigated profusely by neuropsychologists whose main interest so far has been into the executive functions (EFs). EFs are understood as the capacity to act intentionally under the influence of dynamic feedback from the environment. The most typical aspects of EFs include: cognitive inhibition, cognitive elasticity, planning and working memory. Neuropsychologists seem to be in agreement that cognitive inhibition is the most prominent EF deficit in ADHD. Pointing to that are data from children [1,2,3] and adult populations [4].

From a different perspective, researchers in the field of attachment theory [5] have established that ADHD children are classified as insecurely attached much more often than their typically developing peers [6].

It is not clear whether the neuropsychological and the attachment perspectives are looking at two sides of the same coin or at two different coins altogether. In other words, do EF and attachment styles interact to give rise to ADHD symptoms or do they account for different aspects of ADHD?

### 1.1. Executive Functions in ADHD

ADHD was first conceptualized as a “hyperkinetic disease of infancy”. Emphasis was put on a “remarkable motor activity” and resulting problems, such as school disobedience. Attention difficulties, although noticed, were not seen as the main problem area [7]. This changed when Virginia Douglas convincingly demonstrated that hyperkinetic children differed significantly form neurotypical ones not only in terms of behavioral manifestations but also in performance on psychological tests, especially on Continuous Performance Tasks (CPTs) that are still used as a reliable measure of attention [8].

What followed was a multitude of psychological research on ADHD children trying to explain the symptoms of ADHD in terms of cognitive deficits. Meta-analytical studies confirmed that ADHD children perform worse on measures of attention, but also on measures of working memory, inhibition and planning; the latter three grouped together under an umbrella term of executive functions. Impaired inhibition was shown to be characteristic of ADHD children, as compared to typically developing and ones with reading disabilities [3].

As the presence of EF impairment in ADHD children seems to have been well established, current research has shifted to questions about the relationship between executive dysfunctions and ADHD symptoms of inattention and hyperactivity.

One line of thought is that attention problems and executive dysfunctions share a common underlying factor. It has been suggested that poor performance on both types of cognitive tests is due to an increased intra-subject variability in reaction times (ISV) [9]. In other words, ADHD children would not have attention or EF deficits per se but rather their performance would be hampered by irregular disturbances in cognitive processing.

Another line of thought posits that executive functions and attention should be seen as parts of a more complex system of automatic vs. controlled processes [10]. Automatic processes are described as “fast, effortless, autonomous, stereotypic, unavailable to conscious awareness and fairly error-free” while controlled processes are slow and prone to error yet more flexible. In order for an autonomous process to emerge there needs to have been repeated, consciously controlled practice. Recent studies seem to confirm that ADHD children are impaired on both domains [11].

Both lines seem to acknowledge that there may be other, possibly unconscious, factors influencing both attentional and executive performance. Whether they are due to interferences from other systems or to delayed automatization or both is yet unclear. However, there seems to be little discussion about what these factors could be. Some researchers directed their efforts to investigate the relationship between executive functions and attachment in ADHD. Attachment is a likely candidate as it can be described as a set of representations that, on one hand, can influence attentional and executive control, and on the other, is itself an automatic process that needs to be learnt and then modifies behavior without conscious supervision. Their results will be the main focus of this review.

### 1.2. EF Development from Attachment Theory Perspective

Attachment researchers seem to agree with the concept of critical periods in the development of EF. Fonagy and Target [12] claim that self-control is acquired between 14 and 33 months by children who have experienced adequate parenting. Secure attachment is a protective factor against other risk factors of worse continuous performance task scores [13]. In fact, many researchers have been interested in the influence of attachment disorganization on attention [14,15,16].

A securely attached child is able to take advantage of parental scaffolding [17]. Maternal positive affect during play at 10 predicted executive functioning 3, 4 and 5 years later [18].

### 1.3. Environmental Influence on EF in Children with ADHD

Gene–environment interaction is especially important for children with ADHD as they are the ones who often did not experience adequate care in the critical periods. Evidence shows that children who experienced pathogenic care in the first years of life show more ADHD symptoms than the general population [19]. Additionally, Adverse Childhood Experiences (ACEs) account for 10% of EF test score variance in ADHD children [20].

On the other hand, a significant proportion of ADHD children did not experience early deprivation. A comparison of adopted (A-) and not adopted (nA-) children with (-ADHD) and without ADHD (-nADHD) showed that A-ADHD children were the most impaired on EF, followed by nA-ADHD and finally A-nADHD children were the least impaired or not at all [21]. In this case, ADHD explained EF dysfunctions over and above adverse events related to adoption.

### 1.4. Attachment Styles in Children with ADHD

Attachment theory, conceived by Bowlby and operationalized by Ainsworth, posits that a child forms a specific bond with the caregiver in the first years of life. Through that bond, they internalize different kinds of symbols, memories and scripts (or predictable responses to own behavior) that influence later relationships. These internalizations are called internal working models (IWMs). The attachment system is formed within the interval of 6 months and 3 years. Once formed, the pattern of attachment (or attachment style) is rather stable; however, future long-term, close relationships can influence it.

Distributions of attachment styles in typically developing (TDC) and ADHD children are presented in Table 1 [6]:

TDC are mostly securely attached, whereas ADHD children are mostly insecurely attached. Additionally, one-third of ADHD children have a disorganized attachment style, whereas only 6.3% of TDC do.

### 1.5. Aim of This Work

Despite ample research on EF and attachment in ADHD children, we did not find any work that would directly discuss the influence of both variables on ADHD symptoms. So far we have concluded that the possibility of an indirect influence of EFs on ADHD through another variable (such as attachment) is highly likely. Therefore, the goal of this review is to check the database against two hypotheses. Hypothesis 1 (H1): EFs and attachment influence ADHD symptoms independently. Hypothesis 2 (H2): ADHD symptoms are product of a mediation between EFs and attachment.

## 2. Method

### 2.1. Article Selection

We searched the following databases: ProQuest, PubMed, Medline, Wiley, Science Direct, and Scopus. The following search formula was applied: (ADHD OR attention deficit disorder OR attention deficit hyperactivity disorder) AND attention AND (executive functions) AND attachment. The results were limited to peer-reviewed articles in English. We obtained over 5000 results.

The following inclusion criteria were applied:

- At least one research sample underwent some form of ADHD symptom evaluation.
- At least one aspect of EFs was evaluated.
- At least one aspect of attachment was evaluated (attachment style, attachment security).

One exclusion criterion was applied:

- Research sample consisted of adults.

Finally, we included 8 articles in the analysis (see Table 2).

### 2.2. Analysis

We set out to find evidence against both our hypotheses. As our hypotheses were effectively ones about a mediator effect of attachment/EF on ADHD symptoms or lack thereof, we applied criteria for a mediator effect of a variable [30] to set up falsifiability criteria.

**Hypothesis 1** **(H1).** *A study proved that EF and attachment do not influence ADHD symptoms independently, if: (a) lack of influence of both variables on ADHD symptoms was demonstrated; (b) influence of any of the two variables was demonstrated but rendered insignificant after inclusion of the other as a covariate or after inclusion of the interaction effect into the model.*

**Hypothesis 2** **(H2).** *A study proved that EF and attachment do not influence ADHD symptoms through mediation, if: (a) lack of influence of any of the two independent variables was demonstrated; (b) influence of any of the two variables was demonstrated and one of the following: (1) lack of influence of putative mediator on putative predictor was demonstrated or (2) effect of predictor on ADHD symptoms remained significant after inclusion of predictor-mediator interaction.*

## 3. Results

The results of the analysis are presented in Table 3. We concluded that none of the articles refuted H1 and 4 articles refuted H2.

Research refuting H2 was conducted by independent teams. Mean age of the samples falsifying H2 (*Mean* = 7.07; *Standard Deviation* = 1.93) was lower than the one of the other samples (*Mean* = 9.46; *Standard Deviation* = 1.73), although the difference was not statistically significant—*p* > 0.1. In H2-refuting papers, different measures of attachment and similar measures of EF were applied. Research 4 and 5 refuted H2 as it failed to demonstrate the influence of attachment on ADHD symptoms. Research 3 found the influence, but failed to demonstrate a relationship between EFs and attachment. Research 2 found the influence of EFs and attachment on ADHD symptoms as well as a relationship between the predictor and the mediator but the inclusion of the interaction term failed to rid the dependent variables of statistical significance.

All papers found a relationship between EFs and ADHD symptoms. Six papers found a relationship between attachment and ADHD symptoms.

Papers 5 and 6 found a relationship between EFs and both dimensions of ADHD (inattention and impulsiveness/hyperactivity). Paper 6 found an influence of EF and attachment on inattention but only an influence of EF on impulsiveness/hyperactivity.

None of the papers reported a confounding effect of an external variable on EF. Paper 1 reported a confounding effect of early externalizing behaviors on the effect of attachment on ADHD symptoms. Paper 3 reported a confounding effect of general negativity on the effect of attachment on ADHD symptoms.

## 4. Discussion

### 4.1. EFs and Attachment Influence ADHD Symptoms Independently

The conclusion of our analysis is that no existing evidence allows to refute the hypothesis that EFs and attachment influence ADHD symptoms independently. In fact, zero out of eight papers falsified this hypothesis.

Additionally, zero out of eight papers denied the direct influence of EFs on ADHD symptoms. Conversely, the direct influence of attachment on ADHD symptoms finds less support as four out of eight papers failed to demonstrate that such relationship exists.

To further elaborate on that last sentence, it was observed that stimulant medication increased the number of ADHD children categorized as securely attached [31]. The pharmacological intervention increased the percentage of securely attached children from 7% to over 30% and decreased the percentage of disorganized attachment from 15–18% to 0%. This is attributed to drug-enhanced planning abilities. Another explanation for the more frequent insecure/disorganized attachment in ADHD children is a general tendency to negativity [24]. It is therefore possible that the observed attachment insecurity of ADHD children is an artifact produced by EF deficits.

Future research should investigate which aspects of ADHD are explained by EF deficits and which by attachment insecurity. It should also acknowledge the differentiated influence on parent/teacher-reported ADHD symptoms. Lastly, the current state of research warrants a need for an attachment evaluation that is better fitted to the ADHD population.

### 4.2. EF and Attachment Produce ADHD Symptoms through Mediation

The analysis proved the mediator hypothesis to be wrong, at least with the younger ADHD afflicted children. In total, 50% (four out of eight) of the papers excluded the possibility of a mediation. Out of the remaining four, one explained the influence of attachment on ADHD symptoms away by accounting for earlier externalizing behaviors and three did not include the interaction term in their models. This result calls for a modification of the original hypothesis.

A possible reason for the observed lack of influence of attachment on ADHD symptoms may be due to its assessment as a trait. A different approach would assume that on-line attachment representations, rather than attachment style, influence EFs and only then do EF deficits produce ADHD symptoms. By on-line attachment representations we mean attachment representations that interfere with cognitive task performance. Such interferences must be inhibited in order to accomplish a cognitive task. Insecure attachment representations generate anxiety and make more demands on cognitive inhibition resources. In consequence, EFs are reallocated to attachment representation inhibition which results in lower EF test scores. What follows is what we believe to be premises for this line of thought.

The first premise is the recent discovery that the apparent differences on EFs and sustained attention tests between ADHD and TDC are greatly reduced if intra-subject variability in reaction time (ISV) is taken into account [9]. Secondly, ISV has been linked to periodic switches from an attentional neural network to default mode network (DMN) [32]. Thirdly, ISV was found to be related to response searching rather than to stimulus perception [33]. In other words, ISV was found to be related to executive functions, not attention. Fourthly, DMN activity is related to mentalizing, self-reflection and memory consolidation. Securely and insecurely attached people differed in DMN activity while responding to an attachment interview [34].

It is worth noting that research on children not diagnosed with ADHD yielded a mediator effect of planning between disorganized attachment and attention problems and a mediator effect of sustained attention between disorganized attachment and social problems [35].

## 5. Conclusions

This paper reviewed research on influences of EF deficits and attachment on ADHD symptoms. Research was analyzed against its power refute any of two hypotheses: H1: EFs and attachment influence ADHD symptoms independently; H2: ADHD symptoms are a product of a mediation between EFs and attachment. No evidence was found to refute H1; some evidence was found to refute H2. We encountered several methodological problems: validity of standard attachment measures in the ADHD population; assumption that attachment influences ADHD as a trait; concentration on EF test scores without including the confounding effects of intra-subject variability, and paucity of research.

## Figures and Tables

**Table 1:** Distribution of attachment styles in Attention Deficit Hyperactivity Disorder (ADHD) children.

	Secure	Insecure Avoidant	Insecure Ambivalent	Disorganized
No ADHD	52.7%	35.6%	0%	6.3%
ADHD	20.8%	39.6%	6.3%	33.3%

**Table 2:** Articles included in the review.

No	Article	Mean Age of the Sample (SD)	ADHD Diagnosis	Attachment Diagnosis	EF Diagnosis	Main Finding
1	Bohlin, 2012, Disorganized Attachment and Inhibitory Capacity: Predicting Externalizing Problem Behaviors [22]	7.5 (0.42)	Parent and teacher report, a rating scale	Attachment Doll Play Classification System	Go/no-go task, CPT, Stroop task, Knock and tap (NEPSY)	EF and disorganized attachment predicted ADHD independently but disorganized attachment did not when early externalizing problems were controlled for
2	Thorell, 2012, Parent–child attachment and executive functioning in relation to ADHD symptoms in middle childhood [23]	9.5	Teacher’s report (rating scale)	Attachment Doll Play Classification System	Stroop Task, Digit Span (WISC-R), spatial working memory task	Disorganized attachment and EF predict ADHD, but not interaction thereof
3	Scholtens, 2014, ADHD Symptoms and Attachment Representations: Considering the Role of Conduct Problems’, Cognitive Deficits and Narrative Responses in Non-Attachment-Related Story Stems [24]	8.27 (0.96)	Parent and teacher report, ADHD rating scale	A story stem method	Stroop task, Go/No-go task, Children’s size ordering task	Disorganized attachment children had more ADHD symptoms than secure ones. EF correlated with ADHD. No significant differences in terms of EF in attachment groups (additive effects). However, the difference disappeared when negativity was controlled for
4	Forslund, 2016, The heterogeneity of attention-deficit/hyperactivity disorder symptoms and conduct problems: Cognitive inhibition, emotion regulation, emotionality, and disorganized attachment [25]	6.10 (1.7)	Parent report, ADHD rating scale	Separation Anxiety Test	Stroop task, Go/no-go task	Cognitive inhibition predicted ADHD but not disorganized attachment.
5	Lavigne, 2016, Multi-domain Predictors of Attention Deficit/Hyperactivity Disorder Symptoms in Preschool Children: Cross-informant Differences [26]	4.42 (0.43)	Parent and teacher report, Early Childhood Inventory	Attachment Q-Sort	Statue subtest (NEPSY)	Inhibitory Control (IC) and attachment correlated with ADHD. IC but not attachment independently predicted teacher inattentive ADHD and teacher and parent hyperactive/impulsive ADHD
6	Salari, 2017, Neuropsychological Functioning and Attachment Representations in Early School Age as Predictors of ADHD Symptoms in Late Adolescence [27]	8	Parent report, ADHD rating scale	Attachment Doll Play Classification System	Stroop task, Go/no-go task, Digit Span (WISC-R), spatial working memory task	Disorganized attachment and working memory predicted ADHD inattention but only working memory predicted hyperactive/impulsive ADHD
7	Al-Yagon, 2018, Models of child–parent attachment in attention deficit hyperactivity disorder: Links to executive functions [28]	11.45 (0.50)	Conner’s ADHD rating scale, neurological and psychiatric examination	Attachment Security Scale	Behavior Rating Inventory of Executive Functions (teacher report)	EF deficits: ADHD >TDCSecure attachment: to father: TDC > ADHD; to mother: nsEF explained by ADHD and attachment to mother
8	de Maat, 2018, Attention-Deficit Hyperactivity Disorder (ADHD) Symptoms in Children Adopted from Poland and their Atypical Association Patterns: a Bayesian Approach [29]	10.9 (2.7)	Adoptive parents report, ADHD questionnaire	Global Indication List of Attachment	Behavior Rating Inventory of Executive Functions (adoptive parent report)	ADHD symptoms in adopted children more related to attachment and EF than in normal children

Note: ADHD: Attention Deficit Hyperactivity Disorder; EF: executive function; TDC: typically developing children; ns: non significant.

**Table 3:** Results of the analysis.

Research	H1 Falsified	H2 Falsified
1	-	-
2	-	+
3	-	+
4	-	+
5	-	+
6	-	-
7	-	-
8	-	-
